# Supplementary material for: Orienting auditory attention in time: Lateralized alpha power reflects spatio-temporal filtering
Source: Neuroimage. 2021 Mar;228:117711. doi: 10.1016/j.neuroimage.2020.117711 (PMC7903158; doi:10.1016/j.neuroimage.2020.117711)
Supplement: Supplementary file 1 [file mmc1.docx]

**Supplementary Materials**

**
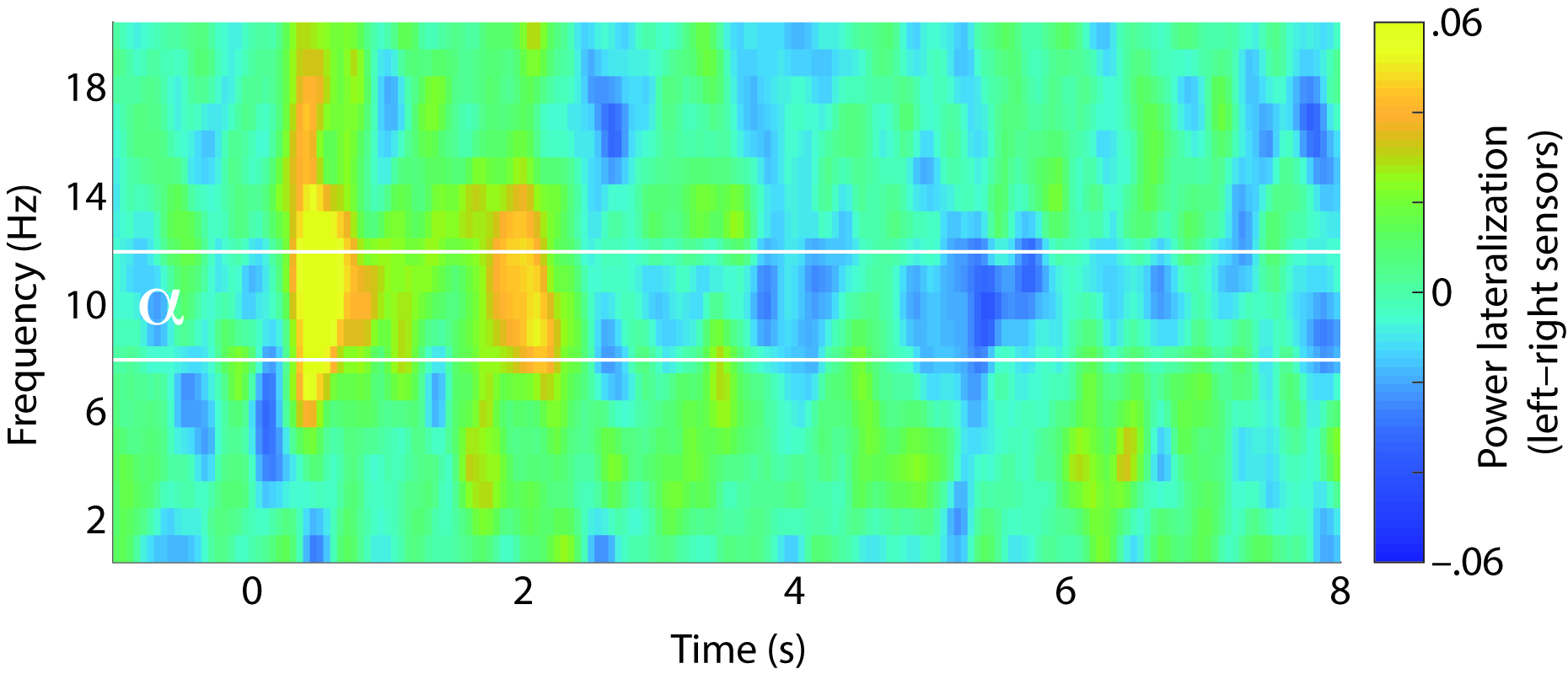
**

**Figure S1.** Time-frequency representation of the lateralization index calculated on oscillatory power (Pow) according to the formula: (Pow_attend-left_ – Pow_attend-right_) / (Pow_attend-left_ + Pow_attend-right_) for all temporal cue conditions (neutral and instructive). The index was calculated at all sensor positions, followed by averaging across left- and right-hemispheric sensors separately, and calculation of the left- minus right-hemisphere difference. Accordingly, positive values indicate higher ipsilateral than contralateral oscillatory power. Hemispheric lateralization of power was strongest in the first third of a trial (~0–2.5 s) in the alpha frequency range (8–12 Hz; highlighted by white horizontal lines), but decreased thereafter and eventually became negative (in agreement with Figure 3E in the main manuscript).

**
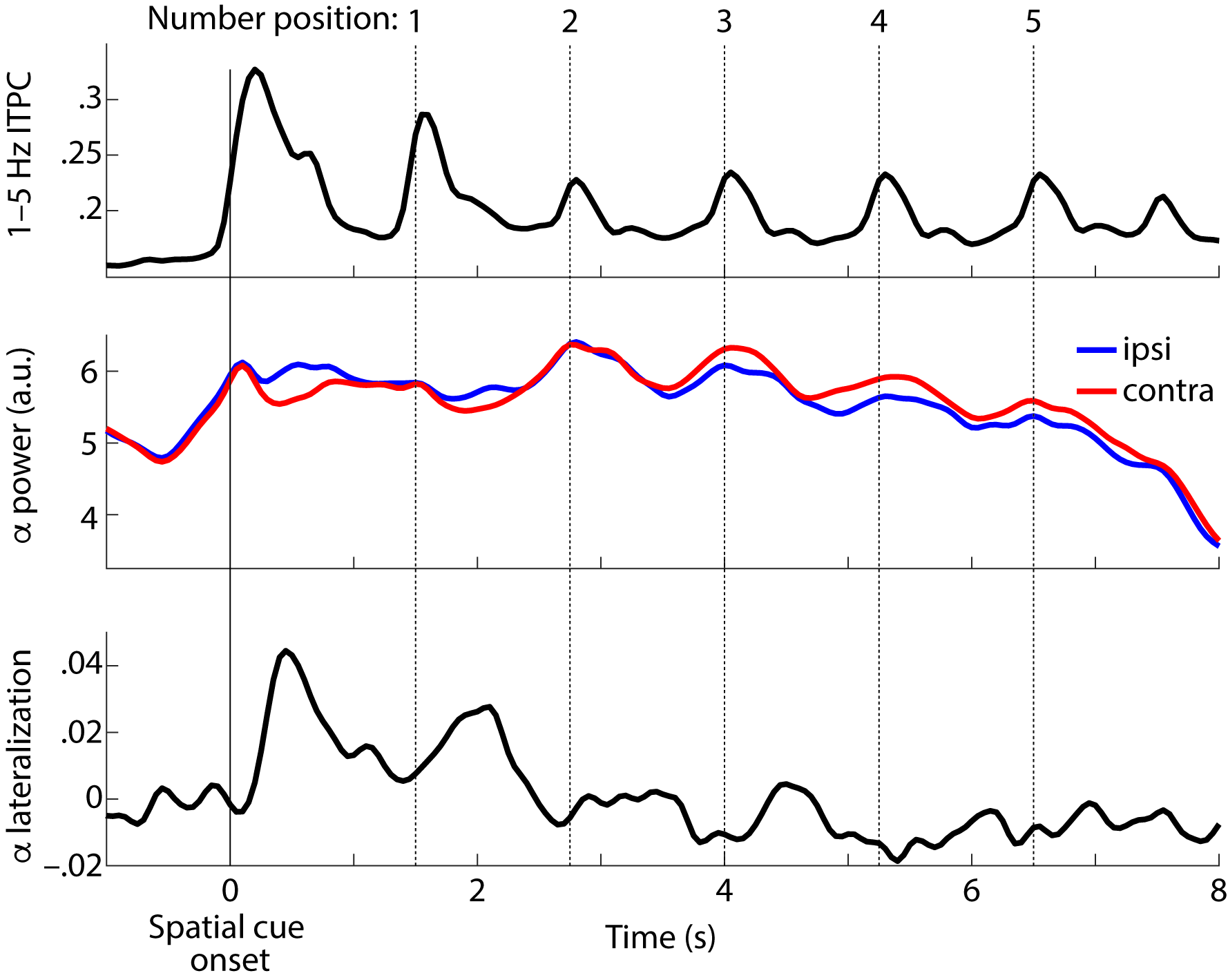
**

**Figure S2.** Top: Time course of low-frequency ITPC (averaged across all *N* = 20 participants, temporal and spatial cue conditions, all 102 combined gradiometer sensors, and frequencies 1–5 Hz). Middle: Time course of absolute alpha power ipsilateral (blue) and contralateral (red) to the focus of spatial attention (averaged across all *N* = 20 participants, temporal and spatial cue conditions, all 102 combined gradiometer sensors, and frequencies 8–12 Hz). Bottom: Time course of the alpha (8–12 Hz) lateralization index, calculated on alpha power (Pow) at sensors ipsilateral (ipsi) versus contralateral (contra) relative to the spatial focus of attention, using the formula: (Pow_ipsi_ – Pow_contra_) / (Pow_ipsi_ + Pow_contra_). Alpha lateralization was averaged across all *N* = 20 participants, and both temporal cue conditions. Vertical solid and dashed lines indicate the onset of the spatial cue and numbers, respectively.

**
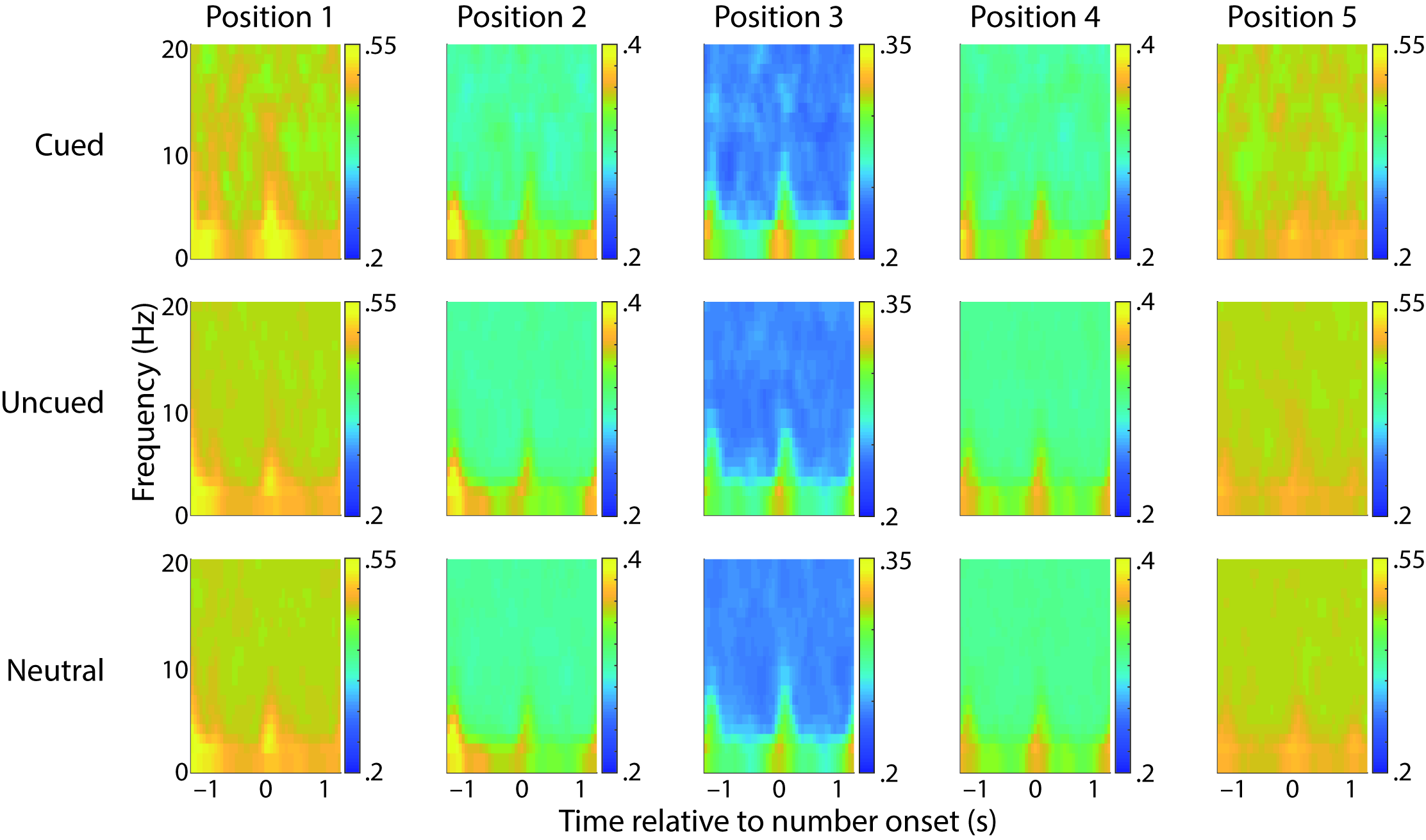
**

**Figure S3.** ITPC for individual number positions (columns) and temporal cueing conditions (rows). ITPC was averaged across all (102) combined gradiometer sensors and *N* = 20 participants. By design, there were less cued than uncued number positions and neutral number positions. For this reason, a subsampling procedure was used that selected for each participant, attended side, and number positions as many uncued and neutral trials as there were cued trials, at random without replacement. This procedure was repeated ten times and ITPC was calculated and averaged across the ten sub-samples. Since ITPC critically depends on trial number, ITPC is generally higher for positions with fewer trials (i.e. positions that were cued less often). However, the sub-sampling procedure allows to compare ITPC across temporal cueing conditions (i.e. within each column). Note different scaling of colour bars.


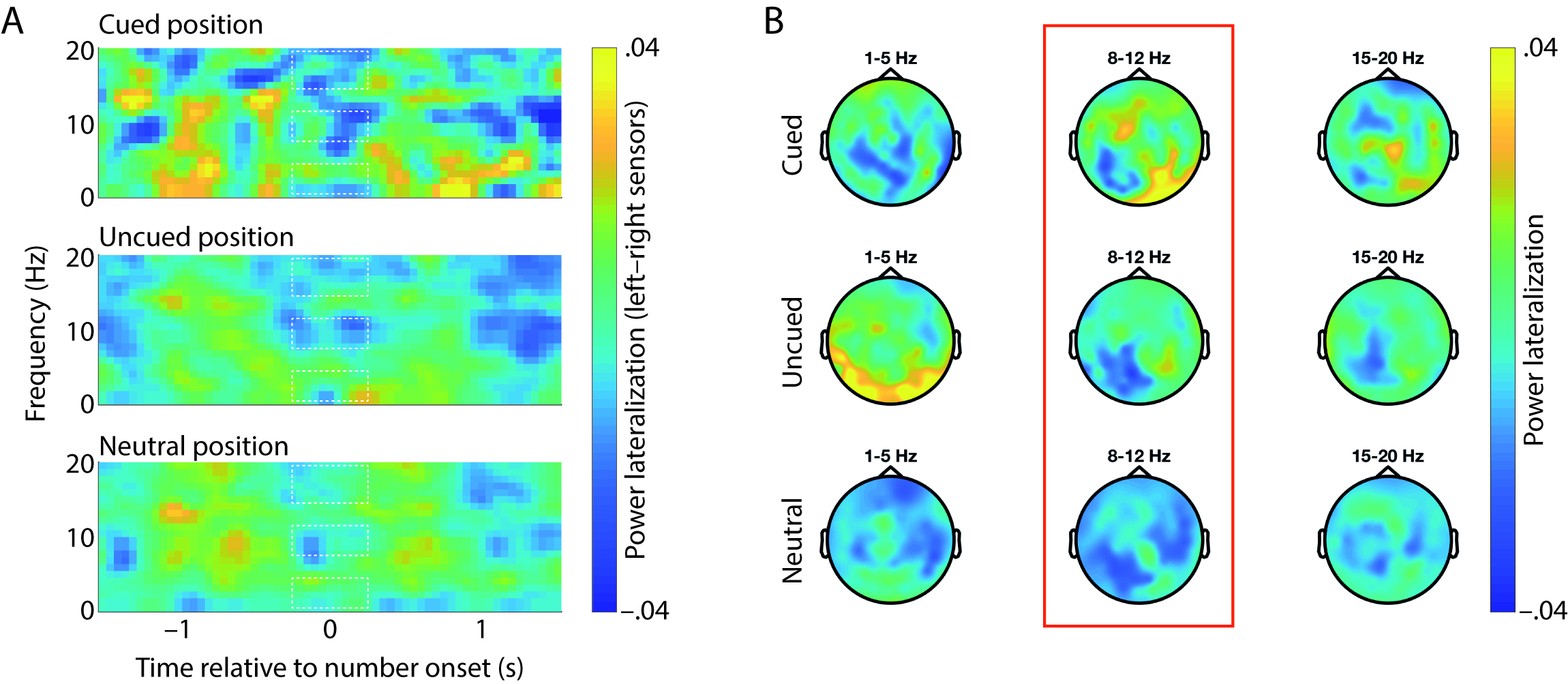


**Figure S4.** (**A**) Same as Figure S1, but for shorter time intervals around cued (top), uncued (middle), and neutral number positions (bottom) within a trial. Positive values indicate higher ipsilateral than contralateral oscillatory power, and vice versa for negative values. Dashed boxes indicate the time interval –0.25 to +0.25 around number onset in the theta (1–5 Hz), alpha (8–12 Hz), and beta (15–20 Hz) frequency bands. (**B**) Topographic maps show power lateralization for different cueing conditions (rows) and frequency bands (columns), calculated according to the formula: (Pow_attend-left_ – Pow_attend-right_) / (Pow_attend-left_ + Pow_attend-right_). In agreement with what is shown in Figure 5 in the main manuscript, alpha lateralization was more negative (i.e. smaller values of the lateralized alpha response on the left versus right hemisphere) in the alpha band at the onset of cued compared with uncued and neutral positions (highlighted by red box). This effect was clearly less obvious in the other frequency bands and can thus be considered alpha-specific.


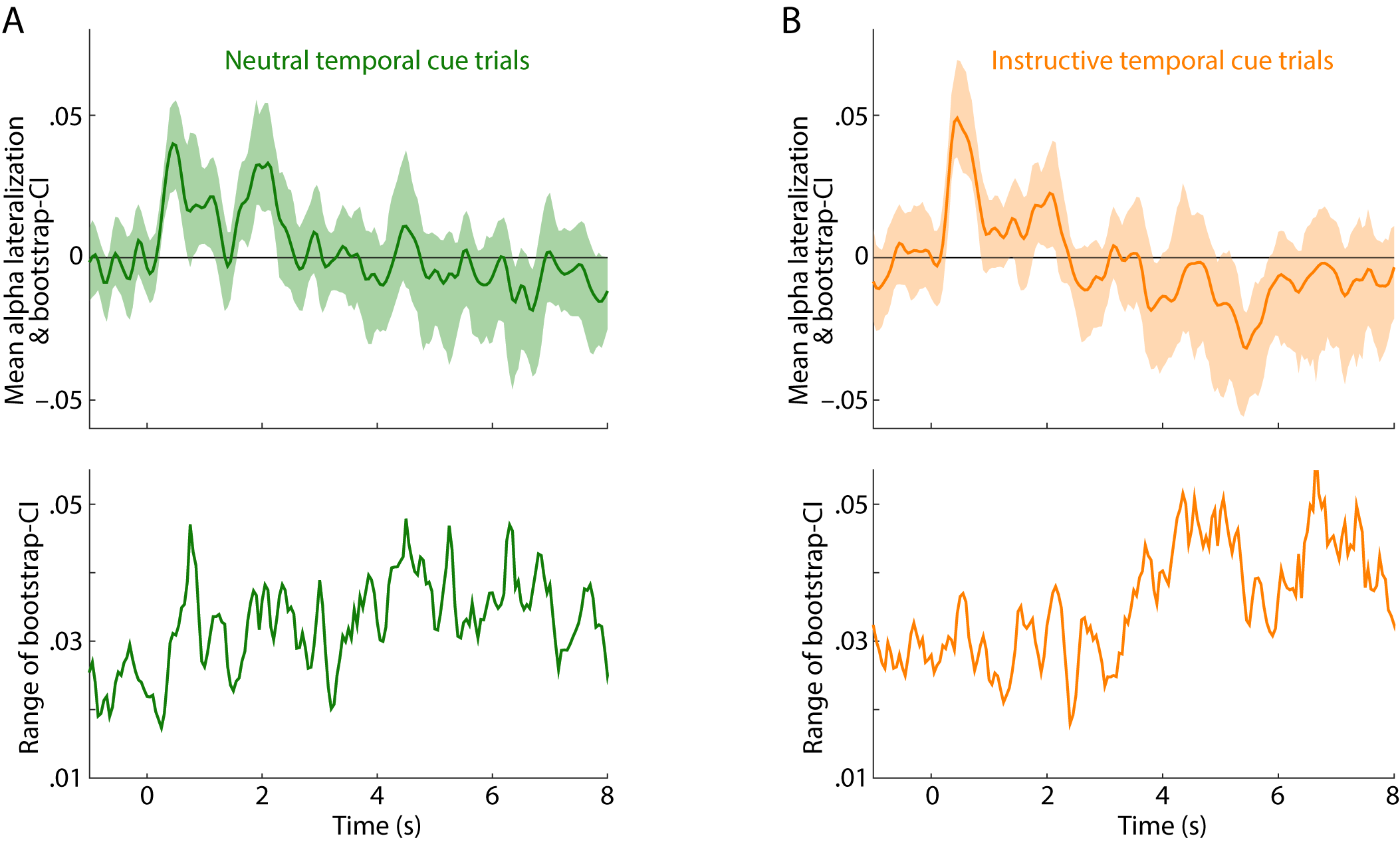


**Figure S5.** (**A**) Top: Solid line and shaded area show the mean alpha lateralization index (similar to Figure 3E in the main manuscript) for neutral temporal cue trials and 95-% bootstrap confidence interval (CI; computed using 1,000 bootstrap samples in the *bootci* function for Matlab), respectively. Bottom: Range of the bootstrap-CI as a function of time. (**B**) Same as (A) but for instructive temporal cue trials. Note that the range of the bootstrap-CI tends to increase later during a trial, which supports the claim that the sign of the late lateralization index should be interpreted with caution.
